# Freehand vs. computer-aided implant surgery: a systematic review and meta-analysis—part 1: accuracy of planned and placed implant position

**DOI:** 10.1186/s40729-025-00622-w

**Published:** 2025-05-02

**Authors:** Joscha G. Werny, Katharina Frank, Shengchi Fan, Keyvan Sagheb, Bilal Al-Nawas, Clement T. Narh, Eik Schiegnitz

**Affiliations:** 1 Department of Oral and Maxillofacial Surgery, Plastic Surgery, University Medical Centre of the Johannes Gutenberg-University, Mainz, Germany; 2 Department of Epidemiology & Biostatistics Fred N. Binka School of Public Health, University of Health and Allied Sciences, PMB31, Ho, Ghana; 3 Oral Surgery and Implantology, Faculty of Medicine and Health Sciences, University of Barcelona, 08907 Barcelona, Spain

## Abstract

**Objectives:**

This systematic review aimed to investigate and compare the accuracy of free-hand and computer-aided implant surgery (CAIS) approaches in dental implant placement.

**Material and methods:**

The PICO question as follows: In patients receiving dental implants, does computer-aided implant surgery superior in accuracy compared to non-computer-aided implant surgery? The primary outcome was angular deviation between the planned and placed position of the implant. An electronic search was made to identify all relevant studies reporting the accuracy of CAIS approaches and freehand for dental implant placement. The data were extracted in the descriptive description, and a meta-analysis of single means was performed to estimate the deviations for each variable using a random-effects model.

**Results:**

Out of 1609 initial articles, 55 were selected for data extraction. The mean value of angular, entry, and apex deviations were 7.46°, 1.56 mm, and 2.22 mm for freehand, 5.94°, 1.13 mm, and 1.43 mm for pilot drill-sCAIS, 2.57°, 0.72 mm, 0.88 mm for fully guided-sCAIS (fg-sCAIS), and 3.67°, 1.01 mm, and 1.36 for dynamic CAIS (dCAIS), respectively. Significant differences were found between the freehand and CAIS approaches (p < 0.04). Fg-sCAIS was significantly more accurate than dCAIS systems at the entry (p < 0.001).

**Conclusions:**

Compared to the freehand approach, both sCAIS and dCAIS improve implant placement accuracy, with angular deviations ranging from 2° to 6°. Detailed planning is crucial for CAIS, particularly for fg-sCAIS, which demonstrated the highest accuracy than others. As apex deviations of 1 to 2 mm have been observed in CAIS approaches, a 2-mm safety margin should be implemented to minimize surgical risks.

## Introduction

Dental implants have proven to be a reliable treatment option, offering long-term stability for treating partial and complete edentulism [1]. The optimal positioning of dental implants is an essential criterion for achieving ideal prosthetic restorations and aesthetic outcomes. Complications such as injuries to anatomical landmarks, compromised esthetics, mechanical issues, and marginal bone loss can be mitigated through well-planned three-dimensional (3D) implant placement [2, 3]. One effective approach to achieve pre-planning implant positioning is the implementation of digital presurgical implant planning. Using computer-aided implant surgery (CAIS) facilitated by either a surgical template or a navigation system, the planned procedure can be accurately transferred to the patient during surgery. Key diagnostic tools include cone beam computed tomography (CBCT) scans, intraoral scans, computer-aided design software (CAD), and computer-aided manufacturing (CAM). This workflow establishes a digital approach that guides drills during osteotomy, facilitating precise implant placement as required for static computer-aided implant surgery (sCAIS) or dynamic computer-aided implant surgery (dCAIS) [4].

In sCAIS, three approaches—pilot-guided, semi-guided, and fully-guided surgery—are differentiated by the design and sleeve diameter in the template, which may influence the surgical outcomes [5–10]. The fully guided approach directs all drills and implant placement, the pilot-guide limits guidance to the initial drill, and the semi-guided approach provides partial guidance but excludes implant insertion. Additionally, the accuracy of template fixation also plays a critical role. It is classified into four types based on the clinical situation: mucosa support, bone support, teeth support, and mixed tissue support.

The application of dCAIS has been utilized in various scenarios, including tumor resection, and zygomatic implant surgery, demonstrating significant benefits with highly accurate assistance [11, 12]. Unlike sCAIS, it relies on a registration and tracking system to guide the surgeon’s performance, allowing for greater flexibility of movement. Navigation systems offer real-time visualization of surgical instruments and the operative field using registration methods with optical tracking technology. However, they appear to be more technically sensitive than sCAIS, requiring the determination of a learning curve for their effective use in implant placement [13–16].

Given the rapid development and widespread adoption of these technologies, along with the growing body of literature on CAIS in recent years, it is crucial to consolidate all available data on the accuracy of various sCAIS and dCAIS approaches. Hence, the primary aim of this systematic review was to evaluate and compare the accuracy of freehand techniques and different CAIS systems in achieving the preoperatively planned implant position.

## Materials and method

The present systematic review followed the guidelines of the “Preferred Reporting Items of Systematic Reviews and Meta-Analysis (PRISMA) [17]. The PICO question was developed: “In patients receiving dental implants, is CAIS superior to freehand surgery in terms of the accuracy of the planned versus placed implant position?” (Table 1). CAIS involves the following key steps: (a) implant planning using digital planning software, (b) determining implant positioning based on 3D radiographic and prosthetic data, and (c) transferring the planned osteotomy position to either a dynamic navigation system or a static surgical template (pilot-, semi- or fully-guided).

**Table 1:** Search tree according to PICO question

PICO—Question	“In patients receiving dental implants, does computer-aided implant surgery have an advantage compared to non-computer-aided implant surgery regarding treatment accuracy, clinical outcome, patient satisfaction, reduce complications surgical time, and treatment costs? „
Population (#1)	P = Fully or partially edentulous patients receiving dental implants a. Dental implant, oral implant, endosseous implant, implant fixture b. MeSH: “Dental implantation”, “Maxillofacial Prosthesis Implantation”, “dental implant”, “Surgery, Oral”
Intervention (#2)	I = Implant placement using computer-aided surgery or non-computer-aided surgery a. free hand, guided dental implant placement, dental surgical guide, dental guided surgery, dental surgical template, computer assisted dental implant, navigation, freehand, fully guided, pilot drill guided, Surgery, Computer-Assisted, Surgery, Oral, dental implant, Maxillofacial Prosthesis Implantation, Dental implantation b. MeSH: “Surgery, Computer-Assisted”, “pilot drill guided”, “fully guided”, “free hand”, “dental navigation”, “computer assisted dental implant”, “guided dental implant placement”, “dental surgical template”, “dental guided surgery”, “dental surgical guide”, “guided dental implant placement”, “dynamic”, “robot”
Comparison (#3)	C = computer-aided or non-computer-aided treatment protocols a. pilot-drill, free hand, non-guided, implant insertion, implant placement, conventional surgery b. MeSH: “Dental implantation”, Endosseous”
Outcome (#4)	O = Accuracy, complications, patient reported outcomes, surgical time, costs of computer-assisted and non-computer-assisted surgery a. Deviation, minutes, operative time, surgical time, mm, costs, complications, patient satisfaction b. MeSH: “efficiency”, “operative time”, “duration of therapy”, “economics”, “Dimensional Measurement Accuracy”, “Intraoperative Complications “, „Postoperative Complications “, “Patient Reported Outcome Measures”
Search combination	#1 AND (#2 OR #3) AND #4

### Eligibility criteria

The following inclusion criteria were defined:

- Only clinical studies (randomized and non-randomized clinical trials (RCTs and non-RCTs), prospective and retrospective observational studies);
- Studies including at least ten patients;
- Studies reported the deviation between the planned and placed implant position;
- Articles written in English or German;
- Studies reporting on conventional and/or digital dental implant placement, including the used systems (software, applications, radiographic assessment, techniques).

The following exclusion criteria were defined:

- Cadaver, animal, and in vitro study;
- Case report, case series, and technical note;
- Patient received zygomatic implant or orthodontic implants;
- Narrative and Systematic reviews;
- Insufficient information on defined criteria.

### Search strategy

A search strategy was developed based on the PICO question and applied for an electronic search in the PubMed and Cochrane databases (Table 1). The search syntax was constructed using combinations of free-text words and Medical Subject Headings [MeSH/EMTREE].

The following search path is exemplary for the PubMed database: (freehand) OR (guided dental implant placement)) OR (dental surgical guide)) OR (dental guided surgery)) OR (dental surgical template)) OR (computer-assisted dental implant)) OR (dental navigation)) OR (freehand)) OR (fully guided)) OR (pilot drill guided)) OR (Surgery, Computer-Assisted)) AND ((((Surgery, Oral) OR (dental implant)) OR (Maxillofacial Prosthesis Implantation)) OR (Dental implantation))).

The publication period of eligible publications was extended from January 1, 2005, to September 6, 2023.

### Study selection and data extraction

Initially, all articles were checked for possible relevance to the topic through their title and, if not applicable, excluded [executed by KF, ES, JW]. Then, the abstracts of the remaining articles were examined for eligibility criteria and possible relevance [conducted by KF, ES, JW]. Finally, in a third stage, the full texts were checked to determine whether they met the inclusion criteria and contained relevant information concerning computer-aided and/or non-computer-aided surgery in terms of complications, surgical time, treatment costs, patient-reported outcomes, and clinical outcome [executed by KF, ES, JW]. This was carried out independently by two reviewers. Disagreements during the selection process were discussed and resolved after each stage; articles were only included if consensus between both authors could be found. No articles were excluded due to non-consensus. Data were collected and filed in an Excel database and EndNote.

The following data were extracted by two independent reviewers [executed by KF, JW] from each relevant full-text article, as far as available, and summarized in a data extraction form:

- Author(s), year of publication, country
- Study design, outcomes
- Time of implant insertion (immediate, delayed, late)
- Time of implant loading (immediate, delayed, late)
- Configuration of the missing teeth (Fully edentulism, partially edentulous)
- Location of the implants (front, premolar, molar, lower/upper jaw)
- Number of patients and implants
- Implant System and Planning software (brand and type)
- Specifications/type of drill guide/navigation system
- Support of drill guide (soft tissue, tooth, bone, pins)
- Flap designs
- Duration/time involved
- Postoperative evaluation
- Complications, Costs, Clinical outcomes, Patient-reported outcomes
- Results and conclusions

The two reviewers repeatedly compared the collected information [executed by KF, JW]. In addition, the corresponding author was requested to provide further written explications if the available data in the article needed to be included or clarified.

### Quality and risk of bias assessment

The quality of the selected observational studies was evaluated according to the STROBE (Strengthening the Reporting of Observational Studies in Epidemiology) statements. CONSORT (Consolidated Standards of Reporting Trials) statements were applied for randomized clinical trials. The bias risk assessment of each study was evaluated using the "Rob 2 Tool". This tool helps to assess the risk of bias in randomized trials [18]. This tool includes algorithms that map responses to signaling questions to a proposed risk-of-bias judgment for each domain in the five domains, including the following: (a) bias arising from the randomization process, (b) bias due to deviations from intended interventions, (c) bias due to missing outcome data, and (d) bias in the measurement of the outcome and bias in the selection of the reported result. The algorithm's specific mappings of each possible combination of answers to the signaling questions (including responses of “No information”) were comprised to grade the risk of bias into the classification low risk of bias, some concerns, or high risk of bias [19]. The “robvis (visualization tool)” web application was used to represent the bias risk assessment of the selected studies graphically.

### Summary measures and synthesis of results

A descriptive analysis of the articles included was performed, and the following data were recorded in a descriptive summary: (1) author, (2) year, (3) country, (4) study design, (5) clinical setting, and (6) details of population, interventions, comparison, and outcomes.

**Fig. 1:**
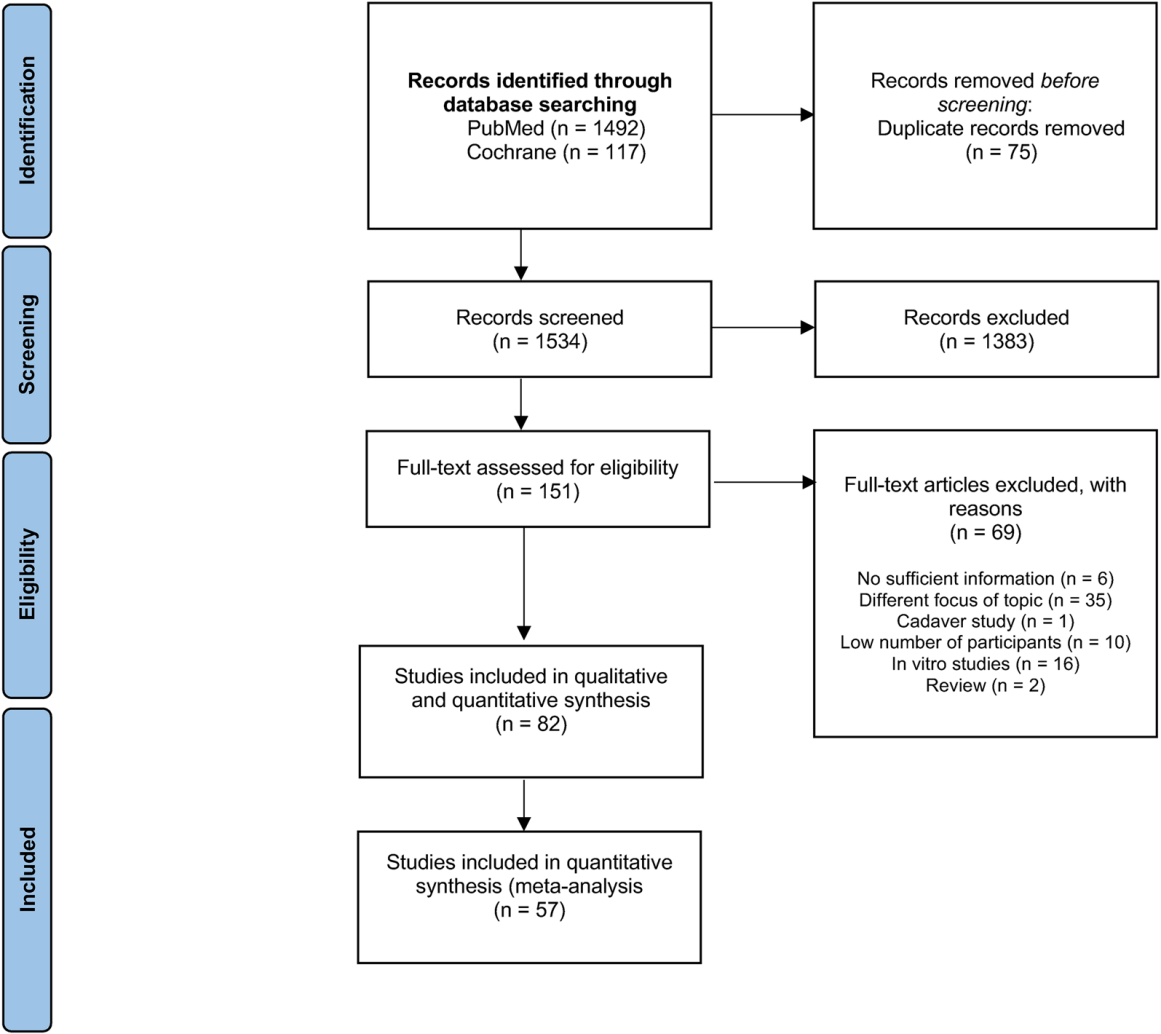
Prisma flow chart

The following outcome variables were analyzed (Fig. 1):

- 2D deviation (horizontal): the deviation of platform and apex between the implant planned and placed position in the *x* and *y* dimensions of space in millimeters (mm). Deviation in depth (*z*-axis) was not considered;
- 3D deviation (global): the deviation of platform and apex between the implant planned and placed position in the three dimensions of space (*x*, *y*, and *z*), in mm;
- Platform deviation (vertical): vertical distance (depth) between the planned position and placed position of the implant platform (*z*-axis), in mm;
- Angulation: angular deviation between the central axes of the planned position and the final position of the implant, in sexadecimal degrees (°)

In the present systematic review, the mean and standard deviation (SD) of the outcome variables were presented based on the weights in each subgroup using the Cochrane Handbook for Systematic Reviews version 6.0 [20]. Using the random effect models in a single mean meta-analysis, we estimated the mean differences of deviation for each outcome variable and a forest plot for all outcomes. Several comparisons were made between freehand and different CAIS approaches. The mean differences were calculated for the planned/placed deviation (Angular deviation, horizontal deviation at Platform and Apex, vertical deviation at Platform and Apex, and global deviation at Platform and Apex). The mean difference, standard error (SE), 95% confidence interval (95% CI), and p-values were reported. We conducted meta-analyses for studies with similar techniques for the same outcomes and positions using the random effect-models. All statistical analyses were performed in Stata 17 and Microsoft Excel, with all statistical significance set at p < 0.05.

## Results

**Fig. 2:**
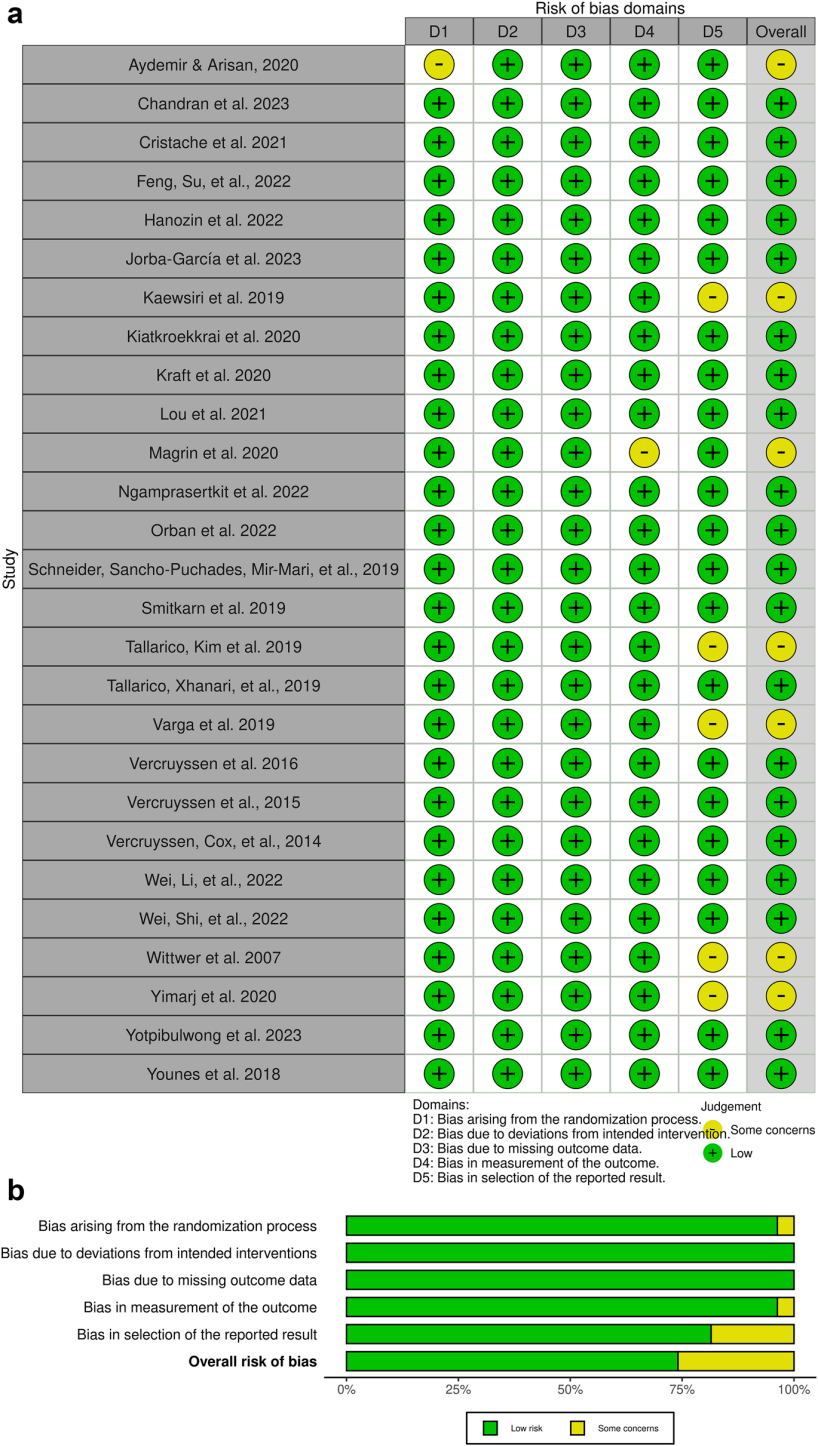
Risk of Bias assessment

Out of 1609 potential articles, 82 were included in the quantitative and qualitative analysis. Sixty-nine reports were excluded after full-text assessment. Figure 2 shows the complete flowchart of the study selection process. The types of studies included for each system can be observed in Table 2. A total of 57 studies were included in this systematic review: 26 RCTs, 22 prospective clinical studies, and nine retrospective clinical studies. Studies unsuitable for the research question were excluded, according to Fig. 2.

**Table 2:** Summary of findings

Study	Country	Study design	Insertion methods	Participants	Implants	Implant manufacturer	Edentulism	Implant placement	Loading procedure	Supporting structure	Pin	Flapless	Conflict of interest
Aydemir & Arisan, 2020	Turkey	RCT, Split mouth	Freehand	30	43	Southern Implants	Partially	NA	NA	NA	No	Flap	No
			dCAIS	30	43								
Block, Emery, Cullum, et al., 2017	USA	Clinical study	freehand	NA	122	NA	NA	NA	NA	NA	No	Flap	No
			dCAIS (pn)	NA	373								
			dCAIS (fn)	NA	212								
Block, Emery, Lank, Ryan et al. 2017	USA	Clinical study	freehand	20	20	NA	partially	NA	NA	NA	No	NA	No
			dCAIS	80	80								
Cassetta et al., 2014	Italy	retrospective clinical study	Fg-sCAIS	18	NA	PRIME	fully	delyed	NA	mucosa	Yes	flapless	No
			Fg-sCAIS	10	NA						No		
Cassetta et al., 2012	Italy	retrospective clinical study	Fg-sCAIS	10	57	Implant System	Partially and fully	NA	Na	mucosa, bone	Yes	Flapless and flap	No
			Fg-sCAIS	10	54						No		
Cassetta et al., 2011	Italy	retrospective clinical study	Fg-sCAIS	10	111	P1H implants	Partially and fully	NA	NA	bone,mucosa and teeth	No	Flapless and flap	
Cassetta, Stefanelli, et al., 2013	Italy	retrospective clinical study	Pg-sCAIS	10	88	NA	Partially and fully	NA	NA	Mucosa	No	Flapless	No
			Pg-sCAIS	2	13					Bone		Flap	
			Pg-sCAIS	2	15					Teeth		Flapless	
Cassetta, Giansanti, et al., 2013	Italy	retrospective clinical study	Fg-sCAIS	10	116	Implant System	Partially and fully	NA	NA	bone,mucosa and teeth	No	Flap and flapless	No
Chandran et al., 2023	India	RCT	Freehand	32	40	Megagen	Partially	immediately	NA	NA	No	NA	No
			Fg-sCAIS	29	40					teeth			
Cristache et al., 2021	Romania, Italy	RCT	Fg-sCAIS (IOS)	25	55	Megagen	Partially	delayed	delayed	teeth	No	flapless	No
			Fg-sCAIS (EOS)	24	56								
Derkens et al., 2019	Netherlands	Clinical study	Fg-sCAIS	66	145	Straumann	Partially	delayed	delayed	teeth	No	Flapless and flap	No
D'Haese et al., 2012	Belgium	Clinical study	Fg-sCAIS	13	78	Densply	Fully	delayed	immediately	Mucosa	Yes	Flapless	No
Di Giacomo et al., 2012	Brazil	Clinical study	Fg-sCAIS	12	60	NA	Fully	NA	Immediate	Mucosa	Yes	Flapless	No
Ersoy et al., 2008	Turkey	Clinical study	Pg-sCAIS	21	94	NA	Partially and fully	NA	NA	bone,mucosa and teeth	No	Flapless and flap	No
Feng, Su, et al., 2022	China	RCT	Fg-sCAIS	20	20	Noble Biocare	Single tooth space	immediate	Immediate or delayed	Teeth	yes	Flapless and flap	No
			dCAIS	20	20					NA	No		
Furhauser et al., 2015	Austria	retrospective clinical study	Pg-sCAIS	27	27	Noble Biocare	Single tooth space	Delayed	NA	Teeth	No	flapless	No
Gelpi et al., 2023	Italy	retrospective clinical study	Pd-sCAIS	15	40	Noble Biocare	Partially	NA	NA	Teeth	No	Flap	No
Geng et al., 2015	China	Clinical study	Pg-sCAIS	NA	29	Straumann	fully	NA	NA	Mucosa	No	Flapless	No
			Fg-sCAIS		30					Mucosa			
			Fg-sCAIS		52					Teeth			
Hanozin et al., 2022	Belgium	RCT	Freehand	9	9	Straumann	Single tooth space	Immediately or delayed	After 10 days	NA	No	Flap	No
			Fg-sCAIS	9	9				immediately	teeth			
Jaemsuwan et al., 2023	Thailand	non-randomized clinical study	Freehand	6	20	Straumann	Partieally and fully	delayed	NA	NA	No	Flap	No
			Fg-sCAIS	4	20						Yes		
			dCAIS	3	20						No		
Jorba-Garcia et al., 2023	Spain	RCT	Freehand	14	22	Straumann and Zimmer	partially	delayed	NA	NA	No	flapless	No
			dCAIS	15	22								
Kaewsiri et al., 2019	Thailand	RCT	Pg-sCAIS	30	30	Straumann	Singel tooth space	Delayed	NA	Teeth	No	Flapless and flap	No
			dCAIS	30	30					NA			
Kiatkroekkrai et al., 2020	Thailand	RCT	Fg-sCAIS (IOS)	30	30	Straumann	Single tooth space	NA	NA	NA	NA	Flapless or flap	No
			Fg-sCAIS (EOS)	30	30								
Kraft et al., 2020	Brazil	RCT	Pd-sCAIS	12	12	Neodent	Single tooth space	immediately	immediately	teeth	No	Flap	No
			Fg-sCAIS	12	12								
Lee et al., 2013	Korea	Clinical study	Fg-sCAIS	48	102	Osstem	Partially and fully	Na	Na	teeth, mucosa	Yes	Flap	No
Lou et al., 2021	China	RCT	Freehand	20	30	Straumann	partially	NA	NA	NA	No	flap	No
			Pd-sCAIS	20	36					teeth			
			Fg-sCAIS	20	33								
Magrin et al., 2020	Brazil	RCT, Split mouth	Freehand	12	12	Straumann	Two single tooth spaces	NA	NA	NA	No	Flap	No
			Pg-sCAIS	12	12					teeth	yes	Flapless	
Ngamprasertkit et al., 2022	Thailand	RCT	Pd-sCAIS	15	15	Novem	Single tooth space	NA	NA	Teeth	No	flap	No
			Fg-sCAIS	15	15								
Nickenig et al., 2010	Germany	Clinical study	Pg-sCAIS	10	23	NA	partially	Na	Na	Teeth	No	flapless	No
Orban et al., 2022	Hungary	RCT	Pg-sCAIS (machine)	20	20	Straumann	Single tooth space	Na	delayed	NA	NA	Flap	No
			Pg-sCAIS (torque wrench)	20	20								
Ozan et al., 2009	Turkey, USA	Clinical study	Pg-sCAIS	NA	30	Zimmer	Partially and fully	NA	NA	Teeth	No	Flap and flapless	No
			Pg-sCAIS	NA	50					Bone			
			Pg-sCAIS	NA	30					Mucosa			
Pellegrino et al., 2019	Italy	Clinical study	dCAIS	NA	NA	Southern Implants	Partially and fully	NA	NA	NA	No	Flap and flapless	No
			dCAIS										
Pettersson et al., 2012	Sweden	Clinical study	Fg-sCAIS	30	139	Nobel Biocare	fully	NA	NA	Mucosa	No	Flapless	No
Sarhan et al., 2021	Egypt	Split mouth clinical study	Pg-sCAIS	12	24	Dentium	fully	NA	NA	Mucosa	Yes	Flapless	No
			Fg-sCAIS	6	24								
Schneider, Sancho-Puchades, Mir-Mari, et al., 2019	Switzerland	RCT	Freehand	26	NA	NA	partially	NA	NA	NA	No	Flap	No
			Pg-sCAIS (SLA)	24	NA								
			Pg-sCAIS (3D print)	23	NA								
Schnutenhaus et al., 2016	Germany	retrospective clinical study	Fg-sCAIS (STG)	12	12	Camlog	Single tooth space	Delayed	Delayed	Teeth	No	Flapless and flap	No
			Fg-sCAIS (DES)	12	12					Teeth and mucosa			
Schnutenhaus et al., 2018	Germany	Clinical study	Fg-sCAIS	12	20	Vita Zahnfabrik	partially	Delayed	Delayed	Teeth	No	Flap and flapless	No
Smitkarn et al., 2019	Thailand	RCT	Freehand	26	30	Straumann	partially	NA	NA	NA	No	Flap	No
			Fg-sCAIS	26	30					teeth			
Stubinger et al., 2014	Switzewrland	Clinical study	Fg-sCAIS	10	44	Desply	fully	Delayed	Delayed	Bone	Yes	Flap	No
Sun et al., 2020	China	Clinical study	Freehand	NA	32	TITC Ltd	Single tooth space	NA	NA	NA	NA	Flap	No
			Pg-sCAIS	NA	32								
			dCAIS	NA	32								
			s- & dCAIS	NA	32								
Tallarico, Kim, et al., 2019	Italy	prospective multicenters clinical study	Pg-sCAIS	16	48	Osstem	Partially and fully	NA	immediately	Teeth	No	Flap or flapless	No
			Fg-sCAIS	23	71								
Tallarico, Xhanari, et al., 2019	Italy	RCT	Fg-sCAIS (EOS)	6	17	Osstem	partially	immediately	immediately	Teeth	Yes	Flap or flapless	No
			Fg-sCAIS (IOS)	6	20								
Testori et al., 2014	Italy	multicenter clinical study	Pg-sCAIS	25	177	NA	NA	NA	NA	bone,mucosa and teeth	No	NA	No
			Pg-sCAIS	NA	NA					Mucosa			
			Pg-sCAIS	NA	NA					Bone			
			Pg-sCAIS	NA	NA					Teeth and mucosa			
Valente et al., 2009	Italy	retrospective clinical study	Pg-sCAIS	25	104	Zimmer and Noble	Partially and fully	NA	NA	bone,mucosa and teeth	No	Flap or flapless	No
Vasak et al., 2011	Austria	Clinical study	Fg-sCAIS	18	86	Nobel Biocare	Fully	NA	Delayed	teeth, mucosa	Yes	Flapless	No
Varga et al., 2020	Hungary	RCT	Freehand	26	55	MultiNeO	partially	NA	NA	NA	No	Flap	No
			Pd-sCAIS	23	49					teeth	No		
			Pg-sCAIS	24	51								
			Fg-sCAIS	28	52								
Vercruyssen et al., 2016	Belgium	RCT	Pg-sCAIS	7	42	Desply	fully	delayed	immediately	mucosa	Yes	flapless	No
									delayed				
Vercruyssen et al., 2015	Belgium	RCT	Freehand	12	51	Desply	fully	delayed	Delayed	NA	No	flap	No
			Pd-sCAIS	12	51						Yes	Flap or flapless	
			Pg-sCAIS	12	55				delayed	mucosa		flapless	
			Pg-sCAIS	12	53					bone		flap	
			Fg-sCAIS	12	52					Mucosa		flapless	
			Fg-sCAIS	12	52					Bone		flap	
Vercruyssen, Cox, et al., 2014	Belgium	RCT	Freehand	12	51	Desply	fully	delayed	Delayed	NA	No	flap	No
			Pd-sCAIS	12	51						Yes	Flap or flapless	
			Pg-sCAIS	12	55				delayed	mucosa		flapless	
			Pg-sCAIS	12	53					bone		flap	
			Fg-sCAIS	12	52					Mucosa		flapless	
			Fg-sCAIS	12	52					Bone		flap	
Verhamme et al., 2017	Netherlands	Clinical study	Fg-sCAIS	12	72	Nobel Biocare	fully	NA	NA	Bone (osteosynthesis)	No	Flapless	No
Vieira et al., 2013	Brazil	Clinical study	Fg-sCAIS	14	62	NA	Fully	Delayed	immediately	Mucosa	Yes	Flapless	No
Wei, Li, et al., 2022	China	RCT, Split mouth	freehand	12	12	Straumann	Single tooth space	immediately	NA	NA	No	Flapless	No
			dCAIS	12	12								
Wei, Shi, et al., 2022	China	RCT	dCAIS	15	20	Straumann (taperded)	Single tooth space	NA	NA	NA	No	NA	No
			dCAIS			Straumann (strait)							
Wittwer et al., 2007	Austria	RCT	dCAIS	8	32	Densply	fully	NA	NA	NA	No	Flapless	No
			dCAIS	8	32								
Yimarj et al., 2020	Thailand	RCT	Fg-sCAIS	15	30	Straumann	Partially	NA	NA	NA	NA	NA	No
			dCAIS	15	30								
Yotpibulwong et al., 2023	Thailand	RCT	Freehand	30	30	Straumann	Single tooth space	NA	NA	NA	No	Flap	No
			Fg-sCAIS	30	30					teeth			
			dCAIS	30	30					NA			
			s- & dCAIS	30	30								
Younes et al., 2018	Belgium	RCT	Freehand	11	26	Desply	Partially	delayed	NA	NA	No	flap	No
			Pd-sCAIS	11	24					teeth		flapless	
			Fg-sCAIS	10	21								

Pd-sCAIS: Pilot-drill guiede Coputer aided implant surgery, Pg-sCAIS: partially guided CAIS, Fg-sCAIS: fully guided CAIS, d-CAIS dynamic navigated CAIS, sCAIS: static CAIS, IOS: Intraoral scan, EOS: extraoral scan, pn: partially navigated, fn: fully navigated, SLA: stereolithographical manufacturing, 3D print: 3D printed manufacturing

Quantitative 57 Studies

Five different implant insertion approaches have been identified, including the free-hand approach and four types of CAIS. Four of them are based on CAIS: pilot-drill-guided (pd-sCAIS), partially-guided (pg-sCAIS), fully-guided (fg-sCAIS), and dCAIS. 18 studies used freehand, with 631 implants in 452 patients; eight used pd-sCAIS, with 278 implants in 120 patients; 19 used pg-sCAIS, with 1222 implants in 519 patients; 35 fg-sCAIS, with 2103 implants in 825 patients, and 15 used dCAIS with 1050 implants in 945 patients.,

Most studies compared the accuracy of a single insertion method with one [9, 21–48], two [49–54], or three alternative techniques [5, 7, 8, 10, 55–58]. Others reported accuracy independently, without direct comparison between different insertion methods [59–94], as presented in Table 3.

**Table 3:** Included Studies separated by the amount of different insertion methods used during the study

Amount of insertion methods Groups	One insertion method	Two different insertion methods	Three different insertion methods	Four different insertion methods
Studies	Abad-Gallegos et al., 2011; Abboud et al., 2012; Cassetta et al., 2014; Cassetta et al., 2012; Cassetta et al., 2011; Cassetta, Stefanelli, et al., 2013; Cristache et al., 2021; D'Haese et al., 2012; Derksen et al., 2019; Di Giacomo et al., 2012; di Torresanto et al., 2014; Ersoy et al., 2008; Furhauser et al., 2015; Gelpi et al., 2023; Kiatkroekkrai et al., 2020; Komiyama et al., 2008; Lee et al., 2013; Marra et al., 2017; Marra et al., 2013; Meloni et al., 2010; Nikzad & Azari, 2010; Orban et al., 2022; Ozan et al., 2009; Ozan et al., 2007; Pellegrino et al., 2019; Pettersson et al., 2012; Pomares, 2010; Schnutenhaus et al., 2016; Schnutenhaus et al., 2018; Stubinger et al., 2014; Tallarico, Xhanari, et al., 2019; Testori et al., 2014; Valente et al., 2009; Vasak et al., 2011; Verhamme et al., 2017; Vieira et al., 2013; Wei, Shi, et al., 2022; Wittwer et al., 2007	Amorfini et al., 2017; Arisan et al., 2010; Block, Emery, Cullum, et al., 2017; Chandran et al., 2023; Elkomy et al., 2021; Feng, Su, et al., 2022; Geng et al., 2015; Hanozin et al., 2022; Jokstad et al., 2018; Jorba-Garcia et al., 2023; Kaewsiri et al., 2019; Kotb Ahmed 2020; Kraft et al., 2020; Kunavisarut et al., 2022; Magrin et al., 2020; Ngamprasertkit et al., 2022; Nickenig et al., 2010; Pozzi et al., 2014; Sancho-Puchades et al., 2019; Sarhan et al., 2021; Schneider et al., 2018; Schneider, Sancho-Puchades, Mir-Mari, et al., 2019; Smitkarn et al., 2019; Tallarico et al., 2018; Tallarico, Kim, et al., 2019; Wei, Li, et al., 2022; Yimarj et al., 2020; Younes et al., 2018; Younes et al., 2019	Afrashtehfar, 2021; Aydemir & Arisan, 2020; Cassetta, Giansanti, et al., 2013; Engkawong et al., 2021; Jaemsuwan et al., 2023; Lou et al., 2021	Bernard et al., 2019; Sun et al., 2020; Varga et al., 2020; Vercruyssen et al., 2015; Vercruyssen, Cox, et al., 2014; Vercruyssen, De Laat, et al., 2014; Vercruyssen, van de Wiele, et al., 2014; Yotpibulwong et al., 2023

25 studies focused on edentulous patients, 23 used sCAIS, two used dCAIS, and one compared both approaches. Regarding comparative studies, two studies compared the sCAIS with/without fixation pins [51, 91]. For the single-arm study, sCAIS with fixation pins were reported in 6 studies [38, 44, 68, 75, 77, 83].

Seventeen studies were conducted in the meta-analysis, shows a mean deviation at implant platform and apex of 1.56 mm (95% CI: 1.38–1.73) and 2.22 mm (95% CI: 1.89–2.56) in freehand approach, 1.13 mm (95% CI: 0.99–1.26) and 1.43 mm (95% CI: 1.21–1.66) in pd-sCAIS, 0.72 mm (95% CI: 0.62–0.81) and 0.86 mm (95% CI: 0.65–1.06) in fg-sCAIS, and 1.01 mm (95% CI: 0.88–1.14) and 1.36 (95% CI: 0.43–2.29) in dCAIS, respectively. However, no studies evaluating pg-sCAIS were included.

**Fig. 3:**
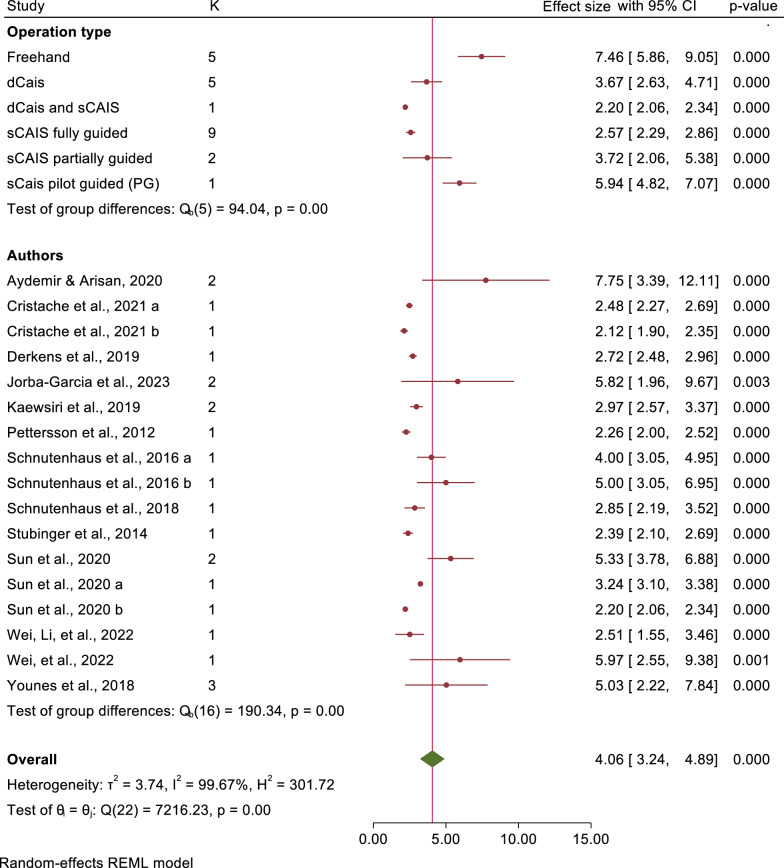
Angular deviation: Combined

**Fig. 4:**
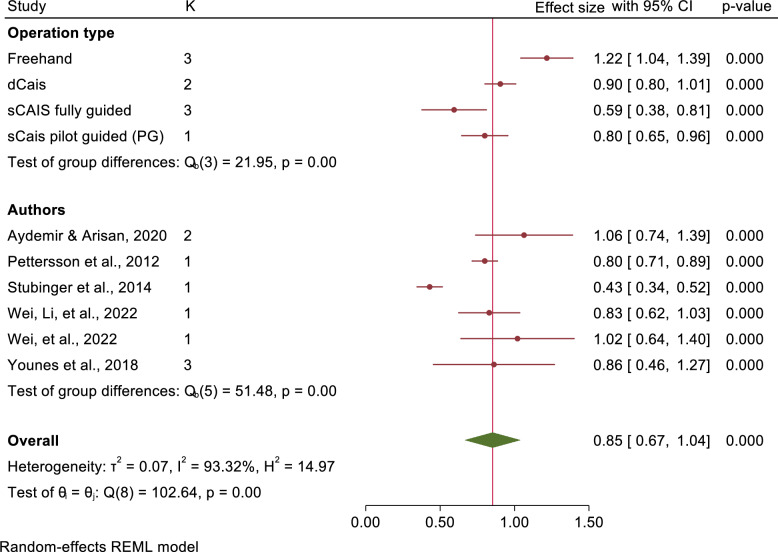
Platform horizontal: Combined

**Fig. 5:**
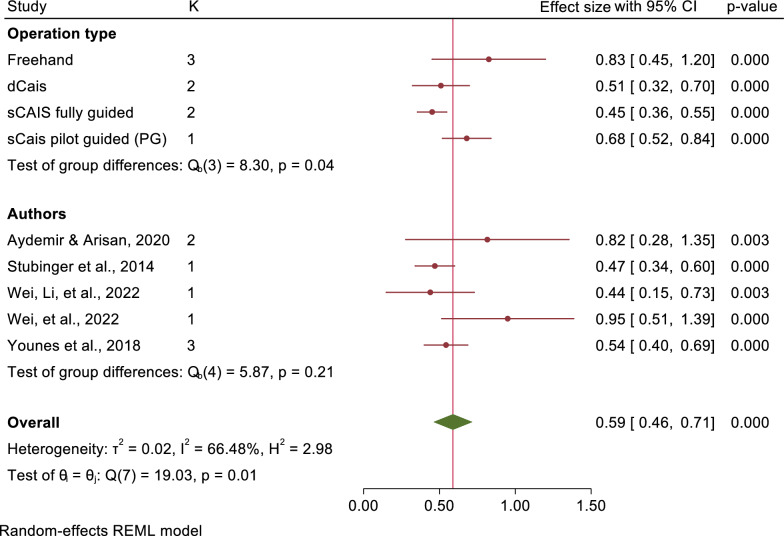
Platform vertical: Combined

**Fig. 6:**
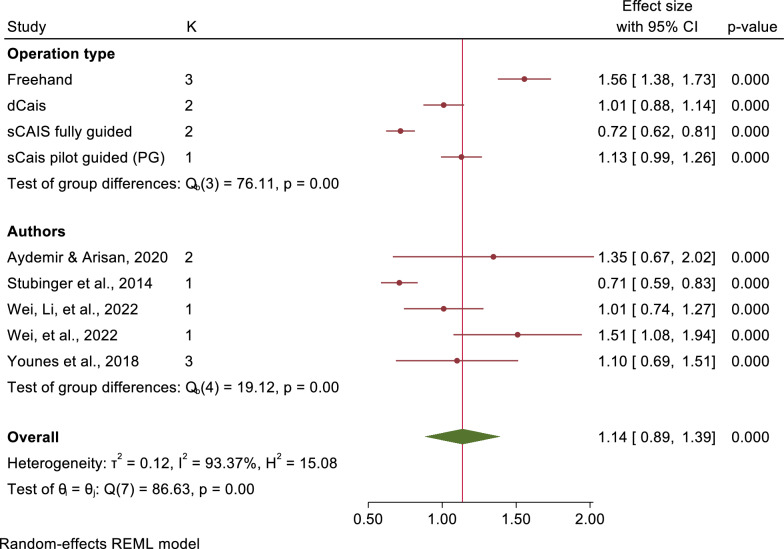
Platform global: Combined

**Fig. 7:**
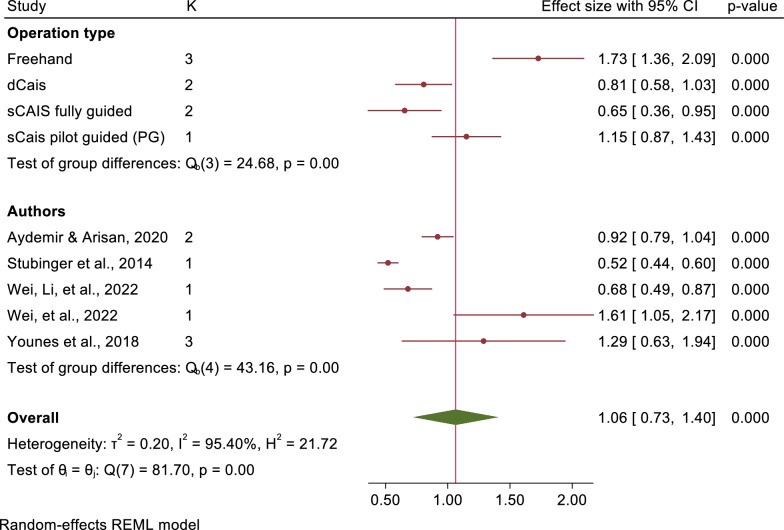
Apex horizontal: Combined

**Fig. 8:**
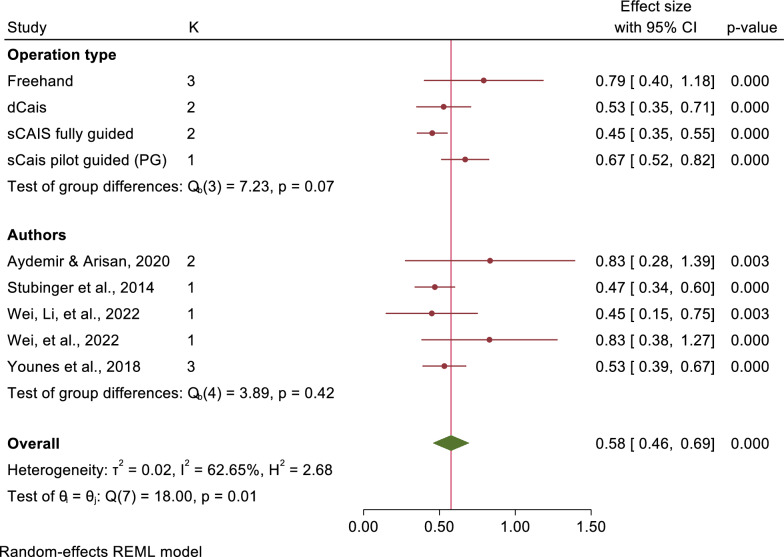
Apex vertical: Combined

**Fig. 9:**
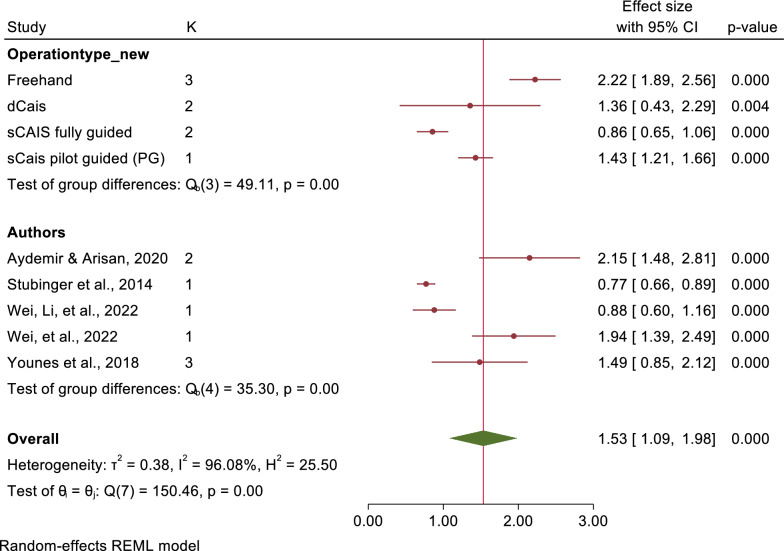
Apex global: Combined

The mean angular deviations were observed 7.46° (95% CI: 5.87–9.05) in freehand approach, 5.94° (95% CI: 4.82–7.07) in pd-sCAIS, 3.72° (95% CI: 2.06–5.38) in pg-sCAIS, 2.57° (95% CI: 2.29–2.86) in fg-sCAIS, 3.67° (95% CI: 2.63–4.71) in dCAIS and 2.20° (95% CI: 2.06–2.34) in a combination of fg-sCAIS and dCAIS (Figs. 3, 4, 5, 6, 7, 8, 9, Tables 4, 5, 6, 7).

**Table 4:** Comparison of freehand and different CAIS methods

Position	Operation type	Operation type	Mean difference (SE) [95% CI]	p-value	Operation type	Operation type	Mean difference (SE) [95% CI]	p-value	Operation type	Operation type	Mean difference (SE) [95% CI]	p-value
	Freehand Mean (SD)	dCAIS Mean (SD)			Freehand Mean (SD)	fg-sCAIS Mean (SD)			Freehand Mean (SD)	Pd-sCAIS Mean (SD)		
Angular deviation	7.43 (1.66)*	3.68 (1.19)	3.75 (0.92) [1.64–5.86]	0.0035*	7.43 (1.66)	2.91 (0.96)	4.52 (0.69) [3.02–(6.03]	0.0000*	7.43 (1.66)	5.94	3.74 (1.32) [0.34–7.15]	0.0368*
Platform horizontal	1.19 (0.14)*	0.88 (0.07)	0.30 (0.11) [0.06–0.67]	0.0742	1.19 (0.14)	0.59 (0.19)	0.59 (0.14) [0.21–0.97]	0.0124*	1.19 (0.14)	0.80	0.39	–
Platform vertical	0.86 (0.30)*	0.50 (0.08)	0.36 (0.23) [0.36–1.09]	0.2088	0.86 (0.30)	0.45 (0.03)	0.41 (0.22) [0.30–1.13]	0.1619	0.86 (0.30)	0.68	0.18	–
Platform global	1.56 (0.13)*	1.01 (0.00)	0.55 (0.09) [0.25–0.85]	0.0102*	1.56 (0.13)	0.72 (0.01)	0.84 (0.09) [0.54–1.14]	0.0031*	1.56 (0.13)	1.13	0.43	–
Apex horizontal	1.63 (0.35)*	0.80 (0.16)	0.83 (0.27) [0.03–1.69]	0.0547	1.63 (0.35)	0.67 (0.21)	0.96 (0.28) [0.06–1.85]	0.0422*	1.63 (0.35)	1.15	0.48	–
Apex vertical	0.82 (0.32)*	0.51 (0.08)	0.31 (0.24) [0.46–1.09]	0.2872	0.82 (0.32)	0.45 (0.03)	0.37 (0.24) [0.39–1.13]	0.2162	0.82 (0.32)	0.67	0.15	–
Apex global	2.19 (0.29)*	1.36 (0.67)	0.84 (0.42) [0.49–2.16]	0.1379	2.19 (0.29)	0.88 (0.15)	1.32 (0.23) [0.58–2.05]	0.0107*	2.19 (0.29)	1.43	0.76	–

*Statistical significance was considered achieved when the p-value was less than 0.05

**Table 5:** Comparison of pd-sCAIS and different CAIS methods

Position	Operation type	Operation type	Mean difference (SE) [95% CI]	p-value	Operation type	Operation type	Mean difference (SE) [95% CI]	p-value	Operation type	Operation type	Mean difference (SE) [95% CI]	p-value
	fg-sCAIS Mean (SD)	Pd-sCAIS Mean (SD)			Pd-sCAIS Mean (SD)	Pg-sCAIS Mean (SD)			dCAIS Mean (SD)	Pd-sCAIS Mean (SD)		
Angular deviation	2.91 (0.96)	5.94*	3.03	–	5.94	3.69 (1.20)	2.25	0.99	3.68 (1.19)	5.94	2.26	–
Platform horizontal	0.59 (0.19)	0.80*	0.21	–	0.80	–	–	–	0.88 (0.07)	0.80	0.08	–
Platform vertical	0.45 (0.03)	0.68*	0.23	–	0.68	–	–	–	0.50 (0.08)	0.68	0.18	–
Platform global	0.72 (0.01)	1.13*	0.41	–	1.13	–	–	–	1.01 (0.00)	1.13	0.12	–
Apex horizontal	0.67 (0.21)	1.15*	0.48	–	1.15	–	–	–	0.80 (0.16)	1.15	0.36	–
Apex vertical	0.45 (0.03)	0.67*	0.22	–	0.67	–	–	–	0.51 (0.08)	0.67	0.16	–
Apex global	0.88 (0.15)	1.43*	0.56	–	1.43	–	–	–	1.36 (0.67)	1.43	0.08	–

*Statistical significance was considered achieved when the p-value was less than 0.05

**Table 6:** Comparison of pg-sCAISand different CAIS methods and freehand

Position	Operation type	Operation type	Mean difference (SE) [95% CI]	p-value	Operation type	Operation type	Mean difference (SE) [95% CI]	p-value	Operation type	Operation type	Mean difference (SE) [95% CI]	p-value
	dCAIS Mean (SD)	pg-sCAIS Mean (SD)			fg-sCAIS Mean (SD)	pg-sCAIS Mean (SD)			Freehand Mean (SD)	pg-sCAIS Mean (SD)		
Angular deviation	3.68 (1.19)	3.69 (1.20)*	0,01 (1.00) [− 2.58–2.57]	0.99	2.91 (0.96)	3.69 (1.20)	0.78 (0.77) [− 2.53–0.97]	0.34	7.43 (1.66)	3.69 (1.20)	3.74 (1.32) [0.34–7.15]	0.0368*
Platform horizontal	0.88 (0.07)	–	–	–	0.59 (0.19)	–	–	–	1.19 (0.14)	–	–	–
Platform vertical	0.50 (0.08)	–	–	–	0.45 (0.03)	–	–	–	0.86 (0.30)	–	–	–
Platform global	1.01 (0.00)	–	–	–	0.72 (0.01)	–	–	–	1.56 (0.13)	–	–	–
Apex horizontal	0.80 (0.16)	–	–	–	0.67 (0.21)	–	–	–	1.63 (0.35)	–	–	–
Apex vertical	0.51 (0.08)	–	–	–	0.45 (0.03)	–	–	–	0.82 (0.32)	–	–	–
Apex global	1.36 (0.67)	–	–	–	0.88 (0.15)	–	–	–	2.19 (0.29)	–	–	–

*Statistical significance was considered achieved when the p-value was less than 0.05

**Table 7:** Comparison of fg-sCAIS and dCAIS

Position	Operation type	Operation type	Mean difference (SE) [95% CI]	p-value
	fg-sCAIS Mean (SD)	dCAIS Mean (SD)		
Angular deviation	2.91 (0.96)	3.68 (1.19)	0.77 (0.58) [− 0.50 to 2.04]	0.20
Platform horizontal	0.59 (0.19)	0.88 (0.07)	0.29 (0.15) [− 0,18 to 0.75]	0.14
Platform vertical	0.45 (0.03)	0.50 (0.08)	0.05 (0.06) [− 0.22 to 0.32]	0.51
Platform global	0.72 (0.01)	1.01 (0.00)	0.29 (0.01) [0.25 to 0.33]	0.0012*
Apex horizontal	0.67 (0.21)	0.80 (0.16)	0.13 (0.19) [− 0.69 to 0.94]	0.58
Apex vertical	0.45 (0.03)	0.51 (0.08)	0.06 (0.06) [− 0.21 to 0.33]	0.44
Apex global	0.88 (0.15)	1.36 (0.67)	0.48 (0.49) [− 1.61 to 2.57]	0.43

*Statistical significance was considered achieved when the p-value was less than 0.05

Significant differences in angular deviation were observed between freehand and all types of CAIS methods. Fg-sCAIS demonstrated significantly greater accuracy at the implant platform compared to both freehand and dCAIS. Additionally, dCAIS was significantly more precise than freehand at the global implant platform. However, only fg-sCAIS showed significantly higher accuracy than freehand for global implant apex deviations. Tables 8 and 9 summarize the mean implant accuracy reported across all studies included in the systematic review, stratified by fixation method. The analyses include both all fixation methods combined and a separate assessment for dental fixation exclusively (Table 10).

**Table 8:** Overall studies, deviation of implant platform, apex, and angular deviation for all fixation methods

Method	Platform global	Apex global	Angular deviation
Freehand	1.76 mm (1.45–2.07)	2.33 mm (2.09–2.56)	7.21° (6.42–8.00)
Pd-sCAIS	1.62 mm (0.93–2.30)	2.09 mm (1.42–2.75)	5.78° (4.25–7.31)
Pg-sCAIS	1.19 mm (1.02–1.35)	1.47 mm (1.31–1.63)	4.23° (3.43–5.03)
fg-sCAIS	1.08 mm (0.92–1.24)	1.44 mm (1.24–1.64)	3.48° (2.89–4.06)
dCAIS	1.03 mm (0.92–1.14)	1.26 mm (1.10–1.42)	3.77° (2.96–4.58)

**Table 9:** Overall studies, deviation of implant platform, apex, and angular deviation for dental fixation only

Method	Platform global	Apex global	Angular deviation
Freehand	1.76 mm (1.45–2.07)	2.33 mm (2.09–2.56)	7.21° (6.42–8.00)
Pd-sCAIS	1.28 mm (1.06–1.50)	1.76 mm (1.53–1.99)	5.12° (4.08–6.15)
Pg-sCAIS	1.24 mm (0.79–1.68)	1.52 mm (1.05–2.00)	3.26° (2.68–3.85)
fg-sCAIS	0.78 mm (0.60–0.96)	1.20 mm (0.90–1.50)	2.58° (2.10–3.05)

**Table 10:** Supporting structure for s-CAIS surgical guides

Comparison of different supporting structure	Bone, mucosa, teeth supported	Only one supporting structure	No comparison of different supporting structures	Supporting structure not reported	Additional use of fixation screws
	(Amorfini et al., 2017; Arisan et al., 2010; Bernard et al., 2019; Cassetta, Giansanti, et al., 2013; Cassetta et al., 2012; Cassetta, Stefanelli, et al., 2013; Geng et al., 2015; Nikzad & Azari, 2010; Ozan et al., 2009; Testori et al., 2014; Vercruyssen, De Laat, et al., 2014; Vercruyssen, van de Wiele, et al., 2014)	(Chandran et al., 2023; Di Giacomo et al., 2012; di Torresanto et al., 2014; Kotb Ahmed 2020; Marra et al., 2013; Meloni et al., 2010; Pettersson et al., 2012; Pomares, 2010; Schnutenhaus et al., 2018; Stubinger et al., 2014; Vieira et al., 2013)	(Abboud et al., 2012; Cassetta et al., 2011; Ersoy et al., 2008; Lee et al., 2013; Tallarico et al., 2018; Testori et al., 2014; Vasak et al., 2011)	(Abad-Gallegos et al., 2011; Afrashtehfar, 2021; Kiatkroekkrai et al., 2020; Komiyama et al., 2008; Ozan et al., 2007; Pozzi et al., 2014; Sancho-Puchades et al., 2019; Schneider et al., 2018)	(Cassetta et al., 2014; Verhamme et al., 2017) (di Torresanto et al., 2014; Marra et al., 2013; Meloni et al., 2010; Pomares, 2010; Pozzi et al., 2014; Tallarico et al., 2018)

## Discussion

CAIS approaches have been developed in implant dentistry to enhance the accuracy and reliability of translating preoperative digital implant planning into precise intraoperative execution, thereby improving clinical outcomes and procedural predictability. The findings of the present review indicate that both sCAIS and dCAIS are reliable techniques, achieving angular deviations that fall within clinically imperceptible ranges (95% CI: 2.06° to 5.38°) across all evaluated methods.

According to previous in-vitro studies, deviation occurs during the initial drilling phase and remains unchanged throughout the subsequent surgical drilling process in fg-sCAIS [95]. For pd-sCAIS and pg-sCAIS, the lack of rigid guidance during drilling increases the likelihood of larger deviations. In contrast, the flexibility in drill positioning during freehand and dCAIS approaches allows for real-time correction of misaligned drills. In particular, the dCAIS navigation system provides feedback that can assist in adjusting drill positioning during the procedure. However, since no studies have investigated planning changes during the operation, this factor remains unaddressed. Nevertheless, the findings of the present meta-analysis indicate that fg-sCAIS achieved the highest accuracy, a trend that has also been observed in in-vitro studies [15].

Significant discrepancies exist between the reported accuracy of clinical and in-vitro studies. In-vitro studies consistently demonstrate substantially higher accuracy for dCAIS compared to clinical studies, with some suggesting that dCAIS outperforms fg-sCAIS [96]. However, the findings of this systematic review reveal the opposite trend in clinical settings, where fg-sCAIS exhibits a greater accuracy. This reversal may be attributed to confounding factors in clinical environments, such as patient movement, swallowing, salivation, bleeding, and restricted mouth opening, which introduce greater variability in dCAIS performance. Notably, fg-sCAIS appears to be less susceptible to these clinical confounders, as its in-vitro accuracy closely aligns with outcomes observed in clinical studies.

Besides the used CAIS approach, the anatomical region of the implant may also play an important role. Some included studies identified significant differences in accuracy between implants placed in the maxilla and mandible. Multiple studies reported that implant placement in the mandible was more accurate than in the maxilla across various methods, including freehand, pd-sCAIS, pg-sCAIS, fg-sCAIS, dCAIS, and combined pg-CAIS with dCAIS [10, 24, 67, 71, 74, 90, 92]. Conversely, other studies noted that while implants in the mandible exhibited greater accuracy in angular deviation, those in the maxilla demonstrated reduced linear deviation during pg-sCAIS [61, 97].

However, implants in the maxilla had significantly less deviation than those in the mandibula in different studies [51, 57]. Also, no significant difference was measured between mandibula and maxilla for implant deviation during pg-sCAIS or fg-sCAIS [45, 62, 69, 82, 85]. Due to the outlined studies' inhomogeneous reporting, a meta-analysis could not be performed on this topic. When the implant position was analyzed in the anterior or posterior region, a more precise result was found in the anterior region, showing significantly lower deviation than in the posterior regions for pd-sCAIS, pg-sCAIS, and fg-sCAIS [45, 67, 71, 90, 91]. Only two studies reported a larger deviation in the anterior region [74] or no significant difference between anterior or posterior inserted implants [65, 85].

A previous systematic review of dCAIS reported an angular deviation of 3.68°, a global coronal deviation of 1.03, and a global apical deviation of 1.34 mm [96]. Similarly, a systematic review focusing on sCAIS found a mean angular deviation of 3.5°, a global coronal deviation of 1.2 mm, and a global apical deviation of 1.4 mm [98]. The findings of the present systematic review align closely with the dCAIS results reported in Jorba-García et al. when considering the dCAIS group. However, some deviations were observed in comparison to the sCAIS results from Tahmaseb et al., likely due to differences in methodological differentiation within the sCAIS group. For fg-sCAIS, the results of the present review demonstrate significantly higher accuracy than the earlier review. Conversely, the results for pg-sCAIS appear consistent with those reported in previous reviews.

CAIS with robotics system assistance has been established within the last few years [99]. To this point, only a small number of clinical studies have been performed. A systematic review of eight clinical studies showed the potential of robotic CAIS [100]. Concerning the implant accuracy, a higher accuracy was achieved than during sCAIS or dCAIS. However, the improvement compared to the results of fg-sCAIS within this review seems marginal. Therefore, further well-designed studies are required to validate and facilitate the broader clinical application of these integrated robotic systems.

Both dCAIS and sCAIS offer the advantage of accurately translating pre-surgical planning into precise implant placement. fg-sCAIS is particularly noted for achieving higher implant placement accuracy and enhancing treatment predictability. However, it necessitates the use of a dedicated surgical guide, which can significantly increase procedural costs. Additionally, sCAIS has other drawbacks, such as extended preoperative planning time, the need for manufacturing and implementing specialized tools, and the potential for technical errors or guide fractures.

Multiple studies have named the 3D printer used to manufacture surgical templates. However, no study has reported on the resin material used for 3D printing. The mechanical properties like the coefficient of elasticity, fragility, printability, and the resulting accuracy of the template may depend on the material used during the printing process. Hence, the printing material could significantly influence the implant accuracy inserted with sCAIS or dCAIS. Further parameters can affect the deviation of different CAIS methods, such as the generation of patient data via digital or conventional impression-taking [64, 72, 87], the flap design [80, 81], the degree of atrophic alveolar [84], the design of the sleeve structure [39–41, 45] or implant design [93], and insertion technique [79]. Additionally, different navigation systems can be used for dCAIS [94]. Further variables influencing the techniques are smoking habit [51], movement during CBCT or implant surgery, offset, and thickness of the static surgical template.

In contrast, dCAIS provides greater intraoperative flexibility, as it does not require a surgical guide and allows real-time adjustments. Despite this advantage, it is associated with a higher risk of implant malposition, primarily due to potential intraoperative deviations from the preoperative plan. This inherent risk highlights the importance of operator expertise and meticulous intraoperative monitoring when using dCAIS.

The maximal mean error in a single study of global deviation at implant platform and apex for freehand was 2.24 mm and 3.60mm, for pd-sCAIS 2.97 mm and 3.40 mm, for pg-sCAIS 2.34 mm and 2.59 mm, for fg-sCAIS 2.05 mm and 2.26 mm, and for dCAIS 1.37 mm and 1.86. Showing a large spread of deviations between the planned/placed implant position. More than 2 mm deviations do not always cause clinical issues, but the larger the deviation, the chance grows. If the available bone does have sufficient quantity and quality freehand, pd-sCAIS and pg-sCAIS can be used. If the bone is not sufficient or exact implant positioning is required, fg-sCAIS and dCAIS can be used to reduce the deviation from the planned position. A safety margin of at least 2 mm should be held to prevent any harm to anatomical structures. In complicated cases, it can be extended up to 3.60 mm.

The limitations of this study regard the external validity, the personal experience of surgeons, the small number of sufficient clinical studies, and the strong representation of few study centers. The results of this meta-analysis may be biased since some of the study groups performed multiple independent studies. Among them were Schneider et al., Vercruyssen et al., Ozan et al., Tallarico et al., Block et al., Wei et al., and Feng et al. Magrin et al., Pimkhaokham et al. and Cassetta et al.. Additionally, the varying experience levels of surgeons in implant dentistry, such as using sCAIS templated and dCAIS navigation systems, may affect clinical implant accuracy. Since there are various possible combinations of 3D printers and resins, the results may differ from those of other adopters. To receive more information on the implant accuracy deviation, we emailed all study groups to ask for missing data (Appendix). After completion, we had only a few studies with sufficient data for the meta-analysis. Additionally, due to a few clinical studies applying pd-sCAIS or pg-sCAIS, no significant differences were found between the pd-sCAIS, pg-sCAIS, and fg-sCAIS protocols.

## Conclusion

Compared to the freehand approach, both sCAIS and dCAIS improve implant placement accuracy, with angular deviations ranging from 2° to 6°. Thorough preoperative planning is crucial for CAIS, especially for fg-sCAIS, which has shown the highest accuracy than others. Given that apex deviations of 1 to 2 mm have been noted in CAIS approaches, a 2-mm safety margin should be implemented to minimize surgical risks. While fg-sCAIS is generally regarded as the clinical gold standard, the limited number of studies on pd-sCAIS and pg-sCAIS prevents definitive conclusions about their efficacy. Future clinical research should focus on evaluating long-term outcomes and cost-effectiveness to develop evidence-based guidelines for the optimal application of CAIS techniques.

## Data Availability

No datasets were generated or analysed during the current study.

## References

[1] Moraschini V, Poubel LA, Ferreira VF, Barboza ES. Evaluation of survival and success rates of dental implants reported in longitudinal studies with a follow-up period of at least 10 years: a systematic review. Int J Oral Maxillofac Surg. 2015;44(3):377–88. 25467739 10.1016/j.ijom.2014.10.023

[2] Buser D, Martin W, Belser UC. Optimizing esthetics for implant restorations in the anterior maxilla: anatomic and surgical considerations. Int J Oral Maxillofac Implants. 2004;19(Suppl):43–61. 15635945

[3] Greenstein G, Cavallaro J, Romanos G, Tarnow D. Clinical recommendations for avoiding and managing surgical complications associated with implant dentistry: a review. J Periodontol. 2008;79(8):1317–29. 18672980 10.1902/jop.2008.070067

[4] Vercruyssen M, Fortin T, Widmann G, Jacobs R, Quirynen M. Different techniques of static/dynamic guided implant surgery: modalities and indications. Periodontol 2000. 2014;66(1):214–27. 25123770 10.1111/prd.12056

[5] Varga E Jr, Antal M, Major L, Kiscsatari R, Braunitzer G, Piffko J. Guidance means accuracy: a randomized clinical trial on freehand versus guided dental implantation. Clin Oral Implants Res. 2020;31(5):417–30. 31958166 10.1111/clr.13578

[6] Vercruyssen M, Coucke W, Naert I, Jacobs R, Teughels W, Quirynen M. Depth and lateral deviations in guided implant surgery: an RCT comparing guided surgery with mental navigation or the use of a pilot-drill template. Clin Oral Implants Res. 2015;26(11):1315–20. 25179585 10.1111/clr.12460

[7] Vercruyssen M, Cox C, Coucke W, Naert I, Jacobs R, Quirynen M. A randomized clinical trial comparing guided implant surgery (bone- or mucosa-supported) with mental navigation or the use of a pilot-drill template. J Clin Periodontol. 2014;41(7):717–23. 24460748 10.1111/jcpe.12231

[8] Yotpibulwong T, Arunjaroensuk S, Kaboosaya B, Sinpitaksakul P, Arksornnukit M, Mattheos N, et al. Accuracy of implant placement with a combined use of static and dynamic computer-assisted implant surgery in single tooth space: a randomized controlled trial. Clin Oral Implants Res. 2023;34(4):330–41. 36756684 10.1111/clr.14043

[9] Younes F, Cosyn J, De Bruyckere T, Cleymaet R, Bouckaert E, Eghbali A. A randomized controlled study on the accuracy of free-handed, pilot-drill guided and fully guided implant surgery in partially edentulous patients. J Clin Periodontol. 2018;45(6):721–32. 29608793 10.1111/jcpe.12897

[10] Sun TM, Lee HE, Lan TH. Comparing accuracy of implant installation with a navigation system (NS), a Laboratory Guide (LG), NS with LG, and Freehand Drilling. Int J Environ Res Public Health. 2020;17(6):2107. 32235745 10.3390/ijerph17062107 PMC7142827

[11] Yang YK, Chan CM, Zhang Q, Xu HR, Niu XH. Computer navigation-aided resection of sacral chordomas. Chin Med J (Engl). 2016;129(2):162–8. 26830986 10.4103/0366-6999.173465 PMC4799542

[12] Wu Y, Tao B, Lan K, Shen Y, Huang W, Wang F. Reliability and accuracy of dynamic navigation for zygomatic implant placement. Clin Oral Implants Res. 2022;33(4):362–76. 35113463 10.1111/clr.13897 PMC9305866

[13] Block MS, Emery RW, Lank K, Ryan J. Implant placement accuracy using dynamic navigation. Int J Oral Maxillofac Implants. 2017;32(1):92–9. 27643585 10.11607/jomi.5004

[14] Feng Y, Yao Y, Yang X. Effect of a dynamic navigation device on the accuracy of implant placement in the completely edentulous mandible: an in vitro study. J Prosthet Dent. 2022;130:731. 35000696 10.1016/j.prosdent.2021.12.012

[15] Marques-Guasch J, Rodriguez-Bauza R, Satorres-Nieto M, Wang HL, Hernandez-Alfaro F, Gargallo-Albiol J. Accuracy of dynamic implant navigation surgery performed by a novice operator. A preliminary study in a cadaveric model. Int J Comput Dent. 2022;0(0):0. 10.3290/j.ijcd.b2588207 35060374

[16] Werny JG, Fan S, Diaz L, Al-Nawas B, Sagheb K, Gielisch M, et al. Evaluation of the accuracy, surgical time, and learning curve of freehand, static, and dynamic computer-assisted implant surgery in an in vitro study. Clin Oral Implants Res. 2025. 10.1111/clr.14403 PMC12066894 39835464

[17] Moher D, Shamseer L, Clarke M, Ghersi D, Liberati A, Petticrew M, et al. Preferred reporting items for systematic review and meta-analysis protocols (PRISMA-P) 2015 statement. Syst Rev. 2015;4(1):1. 25554246 10.1186/2046-4053-4-1 PMC4320440

[18] Sterne JAC, Savovic J, Page MJ, Elbers RG, Blencowe NS, Boutron I, et al. RoB 2: a revised tool for assessing risk of bias in randomised trials. BMJ. 2019;366: l4898. 31462531 10.1136/bmj.l4898

[19] Higgins JPT, Savović J, Page MJ, Elbers RG, Sterne JA. Chapter 8: Assessing risk of bias in a randomized trial. Cochrane Handbook for Systematic Reviews of Interventions 2019. p. 205–28.

[20] Higgins JPT TJ, Chandler J, Cumpston M, Li T, Page MJ, Welch VA (editors) Cochrane Handbook for Systematic Reviews of Interventions version 6.5 (updated August 2024). Available from www.training.cochrane.org/handbook. Cochrane; 2024.

[21] Amorfini L, Migliorati M, Drago S, Silvestrini-Biavati A. Immediately loaded implants in rehabilitation of the maxilla: a two-year randomized clinical trial of guided surgery versus standard procedure. Clin Implant Dent Relat Res. 2017;19(2):280–95. 27790821 10.1111/cid.12459

[22] Arisan V, Karabuda CZ, Ozdemir T. Implant surgery using bone- and mucosa-supported stereolithographic guides in totally edentulous jaws: surgical and post-operative outcomes of computer-aided vs. standard techniques. Clin Oral Implants Res. 2010;21(9):980–8. 20497439 10.1111/j.1600-0501.2010.01957.x

[23] Block MS, Emery RW, Cullum DR, Sheikh A. Implant placement is more accurate using dynamic navigation. J Oral Maxillofac Surg. 2017;75(7):1377–86. 28384461 10.1016/j.joms.2017.02.026

[24] Chandran KRS, Goyal M, Mittal N, George JS. Accuracy of freehand versus guided immediate implant placement: a randomized controlled trial. J Dent. 2023;136: 104620. 37454788 10.1016/j.jdent.2023.104620

[25] Elkomy MM, Khamis MM, El-Sharkawy AM. Clinical and radiographic evaluation of implants placed with fully guided versus partially guided tissue-supported surgical guides: a split-mouth clinical study. J Prosthet Dent. 2021;126(1):58–66. 32768182 10.1016/j.prosdent.2020.05.009

[26] Feng Y, Su Z, Mo A, Yang X. Comparison of the accuracy of immediate implant placement using static and dynamic computer-assisted implant system in the esthetic zone of the maxilla: a prospective study. Int J Implant Dent. 2022;8(1):65. 36512162 10.1186/s40729-022-00464-w PMC9747989

[27] Geng W, Liu C, Su Y, Li J, Zhou Y. Accuracy of different types of computer-aided design/computer-aided manufacturing surgical guides for dental implant placement. Int J Clin Exp Med. 2015;8(6):8442–9. 26309497 PMC4538014

[28] Hanozin B, Li Manni L, Lecloux G, Bacevic M, Lambert F. Digital vs. conventional workflow for one-abutment one-time immediate restoration in the esthetic zone: a randomized controlled trial. Int J Implant Dent. 2022;8(1):7. 35129763 10.1186/s40729-022-00406-6 PMC8821739

[29] Jokstad A, Winnett B, Fava J, Powell D, Somogyi-Ganss E. Investigational clinical trial of a prototype optoelectronic computer-aided navigation device for dental implant surgery. Int J Oral Maxillofac Implants. 2018;33(3):679–92. 29763504 10.11607/jomi.6351

[30] Jorba-Garcia A, Bara-Casaus JJ, Camps-Font O, Sanchez-Garces MA, Figueiredo R, Valmaseda-Castellon E. Accuracy of dental implant placement with or without the use of a dynamic navigation assisted system: a randomized clinical trial. Clin Oral Implants Res. 2023;34(5):438–49. 36794798 10.1111/clr.14050

[31] Kaewsiri D, Panmekiate S, Subbalekha K, Mattheos N, Pimkhaokham A. The accuracy of static vs. dynamic computer-assisted implant surgery in single tooth space: a randomized controlled trial. Clin Oral Implants Res. 2019;30(6):505–14. 31060099 10.1111/clr.13435

[32] Kotb Ahmed EM, Elfar Mahmoud. Esthetic Outcome of Computer-Guided Versus Free-Hand Immediate Implant Placement In Fresh Extraction Sockets in Esthetic Zone, A Randomized Clinical Trial Indian Journal of Public Health Research & Development. 2020;11(12).

[33] Kraft B, Frizzera F, de Freitas RM, de Oliveira G, Marcantonio JE. Impact of fully or partially guided surgery on the position of single implants immediately placed in maxillary incisor sockets: a randomized controlled clinical trial. Clin Implant Dent Relat Res. 2020;22(5):631–7. 32875722 10.1111/cid.12941

[34] Kunavisarut C, Santivitoonvong A, Chaikantha S, Pornprasertsuk-Damrongsri S, Joda T. Patient-reported outcome measures comparing static computer-aided implant surgery and conventional implant surgery for single-tooth replacement: a randomized controlled trial. Clin Oral Implants Res. 2022;33(3):278–90. 34921690 10.1111/clr.13886

[35] Magrin GL, Rafael SNF, Passoni BB, Magini RS, Benfatti CAM, Gruber R, et al. Clinical and tomographic comparison of dental implants placed by guided virtual surgery versus conventional technique: a split-mouth randomized clinical trial. J Clin Periodontol. 2020;47(1):120–8. 31628873 10.1111/jcpe.13211

[36] Ngamprasertkit C, Aunmeungthong W, Khongkhunthian P. The implant position accuracy between using only surgical drill guide and surgical drill guide with implant guide in fully digital workflow: a randomized clinical trial. Oral Maxillofac Surg. 2022;26(2):229–37. 34164754 10.1007/s10006-021-00975-7

[37] Nickenig HJ, Wichmann M, Hamel J, Schlegel KA, Eitner S. Evaluation of the difference in accuracy between implant placement by virtual planning data and surgical guide templates versus the conventional free-hand method—a combined in vivo—in vitro technique using cone-beam CT (Part II). J Craniomaxillofac Surg. 2010;38(7):488–93. 19939691 10.1016/j.jcms.2009.10.023

[38] Pozzi A, Tallarico M, Marchetti M, Scarfo B, Esposito M. Computer-guided versus free-hand placement of immediately loaded dental implants: 1-year post-loading results of a multicentre randomised controlled trial. Eur J Oral Implantol. 2014;7(3):229–42. 25237668

[39] Sancho-Puchades M, Alfaro FH, Naenni N, Jung R, Hammerle C, Schneider D. A randomized controlled clinical trial comparing conventional and computer-assisted implant planning and placement in partially edentulous patients. Part 2: patient related outcome measures. Int J Periodontics Restorative Dent. 2019;39(4):e99–110. 31226187 10.11607/prd.4145

[40] Sarhan MM, Khamis MM, El-Sharkawy AM. Evaluation of the accuracy of implant placement by using fully guided versus partially guided tissue-supported surgical guides with cylindrical versus C-shaped guiding holes: a split-mouth clinical study. J Prosthet Dent. 2021;125(4):620–7. 32389377 10.1016/j.prosdent.2020.02.025

[41] Schneider D, Sancho-Puchades M, Benic GI, Hammerle CH, Jung RE. A randomized controlled clinical trial comparing conventional and computer-assisted implant planning and placement in partially edentulous patients. Part 1: clinician-related outcome measures. Int J Periodontics Restorative Dent. 2018;38:s49–57. 30118533 10.11607/prd.ds2018s

[42] Schneider D, Sancho-Puchades M, Mir-Mari J, Muhlemann S, Jung R, Hammerle C. A randomized controlled clinical trial comparing conventional and computer-assisted implant planning and placement in partially edentulous patients. Part 4: accuracy of implant placement. Int J Periodontics Restorative Dent. 2019;39(4):e111–22. 31226190 10.11607/prd.4147

[43] Smitkarn P, Subbalekha K, Mattheos N, Pimkhaokham A. The accuracy of single-tooth implants placed using fully digital-guided surgery and freehand implant surgery. J Clin Periodontol. 2019;46(9):949–57. 31241782 10.1111/jcpe.13160

[44] Tallarico M, Esposito M, Xhanari E, Caneva M, Meloni SM. Computer-guided vs freehand placement of immediately loaded dental implants: 5-year postloading results of a randomised controlled trial. Eur J Oral Implantol. 2018;11(2):203–13. 29806667

[45] Tallarico M, Kim YJ, Cocchi F, Martinolli M, Meloni SM. Accuracy of newly developed sleeve-designed templates for insertion of dental implants: a prospective multicenters clinical trial. Clin Implant Dent Relat Res. 2019;21(1):108–13. 30592125 10.1111/cid.12704

[46] Wei SM, Li Y, Deng K, Lai HC, Tonetti MS, Shi JY. Does machine-vision-assisted dynamic navigation improve the accuracy of digitally planned prosthetically guided immediate implant placement? A randomized controlled trial. Clin Oral Implants Res. 2022;33(8):804–15. 35652362 10.1111/clr.13961

[47] Yimarj P, Subbalekha K, Dhanesuan K, Siriwatana K, Mattheos N, Pimkhaokham A. Comparison of the accuracy of implant position for two-implants supported fixed dental prosthesis using static and dynamic computer-assisted implant surgery: a randomized controlled clinical trial. Clin Implant Dent Relat Res. 2020;22(6):672–8. 32939934 10.1111/cid.12949

[48] Younes F, Eghbali A, De Bruyckere T, Cleymaet R, Cosyn J. A randomized controlled trial on the efficiency of free-handed, pilot-drill guided and fully guided implant surgery in partially edentulous patients. Clin Oral Implants Res. 2019;30(2):131–8. 30578650 10.1111/clr.13399

[49] Afrashtehfar KI. Conventional free-hand, dynamic navigation and static guided implant surgery produce similar short-term patient-reported outcome measures and experiences. Evid Based Dent. 2021;22(4):143–5. 34916642 10.1038/s41432-021-0216-9

[50] Aydemir CA, Arısan V. Accuracy of dental implant placement via dynamic navigation or the freehand method: a split-mouth randomized controlled clinical trial. Clin Oral Implants Res. 2020;31(3):255–63. 31829457 10.1111/clr.13563

[51] Cassetta M, Giansanti M, Di Mambro A, Stefanelli LV. Accuracy of positioning of implants inserted using a mucosa-supported stereolithographic surgical guide in the edentulous maxilla and mandible. Int J Oral Maxillofac Implants. 2014;29(5):1071–8. 25216132 10.11607/jomi.3329

[52] Engkawong S, Mattheos N, Pisarnturakit PP, Pimkhaokham A, Subbalekha K. Comparing patient-reported outcomes and experiences among static, dynamic computer-aided, and conventional freehand dental implant placement: a randomized clinical trial. Clin Implant Dent Relat Res. 2021;23(5):660–70. 34231956 10.1111/cid.13030

[53] Jaemsuwan S, Arunjaroensuk S, Kaboosaya B, Subbalekha K, Mattheos N, Pimkhaokham A. Comparison of the accuracy of implant position among freehand implant placement, static and dynamic computer-assisted implant surgery in fully edentulous patients: a non-randomized prospective study. Int J Oral Maxillofac Surg. 2023;52(2):264–71. 35752531 10.1016/j.ijom.2022.05.009

[54] Lou F, Rao P, Zhang M, Luo S, Lu S, Xiao J. Accuracy evaluation of partially guided and fully guided templates applied to implant surgery of anterior teeth: a randomized controlled trial. Clin Implant Dent Relat Res. 2021;23(1):117–30. 33528110 10.1111/cid.12980

[55] Bernard L, Vercruyssen M, Duyck J, Jacobs R, Teughels W, Quirynen M. A randomized controlled clinical trial comparing guided with nonguided implant placement: a 3-year follow-up of implant-centered outcomes. J Prosthet Dent. 2019;121(6):904–10. 30732920 10.1016/j.prosdent.2018.09.004

[56] Vercruyssen M, Laleman I, Jacobs R, Quirynen M. Computer-supported implant planning and guided surgery: a narrative review. Clin Oral Implants Res. 2015;26(Suppl 11):69–76. 26385623 10.1111/clr.12638

[57] Vercruyssen M, De Laat A, Coucke W, Quirynen M. An RCT comparing patient-centred outcome variables of guided surgery (bone or mucosa supported) with conventional implant placement. J Clin Periodontol. 2014;41(7):724–32. 24708422 10.1111/jcpe.12257

[58] Vercruyssen M, van de Wiele G, Teughels W, Naert I, Jacobs R, Quirynen M. Implant- and patient-centred outcomes of guided surgery, a 1-year follow-up: an RCT comparing guided surgery with conventional implant placement. J Clin Periodontol. 2014;41(12):1154–60. 25197015 10.1111/jcpe.12305

[59] Abad-Gallegos M, Gomez-Santos L, Sanchez-Garces MA, Pinera-Penalva M, Freixes-Gil J, Castro-Garcia A, et al. Complications of guided surgery and immediate loading in oral implantology: a report of 12 cases. Med Oral Patol Oral Cir Bucal. 2011;16(2):e220–4. 20711144 10.4317/medoral.16.e220

[60] Abboud M, Wahl G, Calvo-Guirado JL, Orentlicher G. Application and success of two stereolithographic surgical guide systems for implant placement with immediate loading. Int J Oral Maxillofac Implants. 2012;27(3):634–43. 22616058

[61] Cassetta M, Stefanelli LV, Giansanti M, Calasso S. Accuracy of implant placement with a stereolithographic surgical template. Int J Oral Maxillofac Implants. 2012;27(3):655–63. 22616060

[62] Cassetta M, Stefanelli LV, Giansanti M, Di Mambro A, Calasso S. Depth deviation and occurrence of early surgical complications or unexpected events using a single stereolithographic surgi-guide. Int J Oral Maxillofac Surg. 2011;40(12):1377–87. 22001378 10.1016/j.ijom.2011.09.009

[63] Cassetta M, Stefanelli LV, Giansanti M, Di Mambro A, Calasso S. Accuracy of a computer-aided implant surgical technique. Int J Periodontics Restorative Dent. 2013;33(3):317–25. 23593625 10.11607/prd.1019

[64] Cristache CM, Burlibasa M, Tudor I, Totu EE, Di Francesco F, Moraru L. Accuracy, labor-time and patient-reported outcomes with partially versus fully digital workflow for flapless guided dental implants insertion-a randomized clinical trial with one-year follow-up. J Clin Med. 2021;10(5):1102. 33800946 10.3390/jcm10051102 PMC7961841

[65] D’Haese J, Van De Velde T, Elaut L, De Bruyn H. A prospective study on the accuracy of mucosally supported stereolithographic surgical guides in fully edentulous maxillae. Clin Implant Dent Relat Res. 2012;14(2):293–303. 19906267 10.1111/j.1708-8208.2009.00255.x

[66] Derksen W, Wismeijer D, Flugge T, Hassan B, Tahmaseb A. The accuracy of computer-guided implant surgery with tooth-supported, digitally designed drill guides based on CBCT and intraoral scanning. A prospective cohort study. Clin Oral Implants Res. 2019;30(10):1005–15. 31330566 10.1111/clr.13514

[67] Di Giacomo GA, da Silva JV, da Silva AM, Paschoal GH, Cury PR, Szarf G. Accuracy and complications of computer-designed selective laser sintering surgical guides for flapless dental implant placement and immediate definitive prosthesis installation. J Periodontol. 2012;83(4):410–9. 21819249 10.1902/jop.2011.110115

[68] di Torresanto VM, Milinkovic I, Torsello F, Cordaro L. Computer-assisted flapless implant surgery in edentulous elderly patients: a 2-year follow up. Quintessence Int. 2014;45(5):419–29. 24634906 10.3290/j.qi.a31534

[69] Ersoy AE, Turkyilmaz I, Ozan O, McGlumphy EA. Reliability of implant placement with stereolithographic surgical guides generated from computed tomography: clinical data from 94 implants. J Periodontol. 2008;79(8):1339–45. 18672982 10.1902/jop.2008.080059

[70] Furhauser R, Mailath-Pokorny G, Haas R, Busenlechner D, Watzek G, Pommer B. Esthetics of flapless single-tooth implants in the anterior maxilla using guided surgery: association of three-dimensional accuracy and pink esthetic score. Clin Implant Dent Relat Res. 2015;17(Suppl 2):e427–33. 25346154 10.1111/cid.12264

[71] Gelpi F, Modena N, Poscolere A, Bernardello F, Torroni L, De Santis D. Accuracy of computer-guided implantology with pilot drill surgical guide: retrospective 3D radiologic investigation in partially edentulous patients. Medicina (Kaunas). 2023;59(4):738. 37109696 10.3390/medicina59040738 PMC10142633

[72] Kiatkroekkrai P, Takolpuckdee C, Subbalekha K, Mattheos N, Pimkhaokham A. Accuracy of implant position when placed using static computer-assisted implant surgical guides manufactured with two different optical scanning techniques: a randomized clinical trial. Int J Oral Maxillofac Surg. 2020;49(3):377–83. 31543382 10.1016/j.ijom.2019.08.019

[73] Komiyama A, Klinge B, Hultin M. Treatment outcome of immediately loaded implants installed in edentulous jaws following computer-assisted virtual treatment planning and flapless surgery. Clin Oral Implants Res. 2008;19(7):677–85. 18565011 10.1111/j.1600-0501.2008.01538.x

[74] Lee JH, Park JM, Kim SM, Kim MJ, Lee JH, Kim MJ. An assessment of template-guided implant surgery in terms of accuracy and related factors. J Adv Prosthodont. 2013;5(4):440–7. 24353883 10.4047/jap.2013.5.4.440 PMC3865200

[75] Marra R, Acocella A, Rispoli A, Sacco R, Ganz SD, Blasi A. Full-mouth rehabilitation with immediate loading of implants inserted with computer-guided flap-less surgery: a 3-year multicenter clinical evaluation with oral health impact profile. Implant Dent. 2013;22(5):444–52. 24021974 10.1097/ID.0b013e31829f1f7f

[76] Marra R, Acocella A, Alessandra R, Ganz SD, Blasi A. Rehabilitation of full-mouth edentulism: immediate loading of implants inserted with computer-guided flapless surgery versus conventional dentures: a 5-year multicenter retrospective analysis and OHIP Questionnaire. Implant Dent. 2017;26(1):54–8. 27749520 10.1097/ID.0000000000000492

[77] Meloni SM, De Riu G, Pisano M, Cattina G, Tullio A. Implant treatment software planning and guided flapless surgery with immediate provisional prosthesis delivery in the fully edentulous maxilla. A retrospective analysis of 15 consecutively treated patients. Eur J Oral Implantol. 2010;3(3):245–51. 20847994

[78] Nikzad S, Azari A. Custom-made radiographic template, computed tomography, and computer-assisted flapless surgery for treatment planning in partial edentulous patients: a prospective 12-month study. J Oral Maxillofac Surg. 2010;68(6):1353–9. 20231051 10.1016/j.joms.2009.04.108

[79] Orban K, Varga E Jr, Windisch P, Braunitzer G, Molnar B. Accuracy of half-guided implant placement with machine-driven or manual insertion: a prospective, randomized clinical study. Clin Oral Investig. 2022;26(1):1035–43. 34401946 10.1007/s00784-021-04087-0 PMC8791874

[80] Ozan O, Turkyilmaz I, Yilmaz B. A preliminary report of patients treated with early loaded implants using computerized tomography-guided surgical stents: flapless versus conventional flapped surgery. J Oral Rehabil. 2007;34(11):835–40. 17919250 10.1111/j.1365-2842.2007.01772.x

[81] Pellegrino G, Taraschi V, Andrea Z, Ferri A, Marchetti C. Dynamic navigation: a prospective clinical trial to evaluate the accuracy of implant placement. Int J Comput Dent. 2019;22(2):139–47. 31134220

[82] Pettersson A, Komiyama A, Hultin M, Nasstrom K, Klinge B. Accuracy of virtually planned and template guided implant surgery on edentate patients. Clin Implant Dent Relat Res. 2012;14(4):527–37. 20491812 10.1111/j.1708-8208.2010.00285.x

[83] Pomares C. A retrospective study of edentulous patients rehabilitated according to the “all-on-four” or the “all-on-six” immediate function concept using flapless computer-guided implant surgery. Eur J Oral Implantol. 2010;3(2):155–63. 20623040

[84] Schnutenhaus S, Edelmann C, Rudolph H, Luthardt RG. Retrospective study to determine the accuracy of template-guided implant placement using a novel nonradiologic evaluation method. Oral Surg Oral Med Oral Pathol Oral Radiol. 2016;121(4):e72–9. 26972545 10.1016/j.oooo.2015.12.012

[85] Schnutenhaus S, von Koenigsmarck V, Blender S, Ambrosius L, Luthardt RG, Rudolph H. Precision of sleeveless 3D drill guides for insertion of one-piece ceramic implants: a prospective clinical trial. Int J Comput Dent. 2018;21(2):97–105. 29967902

[86] Stubinger S, Buitrago-Tellez C, Cantelmi G. Deviations between placed and planned implant positions: an accuracy pilot study of skeletally supported stereolithographic surgical templates. Clin Implant Dent Relat Res. 2014;16(4):540–51. 23167722 10.1111/cid.12019

[87] Tallarico M, Xhanari E, Kim YJ, Cocchi F, Martinolli M, Alushi A, et al. Accuracy of computer-assisted template-based implant placement using conventional impression and scan model or intraoral digital impression: a randomised controlled trial with 1 year of follow-up. Int J Oral Implantol (Berl). 2019;12(2):197–206. 31090750

[88] Testori T, Robiony M, Parenti A, Luongo G, Rosenfeld AL, Ganz SD, et al. Evaluation of accuracy and precision of a new guided surgery system: a multicenter clinical study. Int J Periodontics Restorative Dent. 2014;34(Suppl 3):s59-69. 24956092 10.11607/prd.1279

[89] Valente F, Schiroli G, Sbrenna A. Accuracy of computer-aided oral implant surgery: a clinical and radiographic study. Int J Oral Maxillofac Implants. 2009;24(2):234–42. 19492638

[90] Vasak C, Watzak G, Gahleitner A, Strbac G, Schemper M, Zechner W. Computed tomography-based evaluation of template (NobelGuide)-guided implant positions: a prospective radiological study. Clin Oral Implants Res. 2011;22(10):1157–63. 21244498 10.1111/j.1600-0501.2010.02070.x

[91] Verhamme LM, Meijer GJ, Soehardi A, Berge SJ, Xi T, Maal TJJ. An accuracy study of computer-planned implant placement in the augmented maxilla using osteosynthesis screws. Int J Oral Maxillofac Surg. 2017;46(4):511–7. 27887876 10.1016/j.ijom.2016.10.013

[92] Vieira DM, Sotto-Maior BS, Barros CA, Reis ES, Francischone CE. Clinical accuracy of flapless computer-guided surgery for implant placement in edentulous arches. Int J Oral Maxillofac Implants. 2013;28(5):1347–51. 24066327 10.11607/jomi.3156

[93] Wei SM, Shi JY, Qiao SC, Zhang X, Lai HC, Zhang XM. Accuracy and primary stability of tapered or straight implants placed into fresh extraction socket using dynamic navigation: a randomized controlled clinical trial. Clin Oral Investig. 2022;26(3):2733–41. 34797431 10.1007/s00784-021-04247-2

[94] Wittwer G, Adeyemo WL, Schicho K, Birkfellner W, Enislidis G. Prospective randomized clinical comparison of 2 dental implant navigation systems. Int J Oral Maxillofac Implants. 2007;22(5):785–90. 17974114

[95] Raabe C, Abou-Ayash S, Yilmaz B, Surbek FJ, Chappuis V, Couso-Queiruga E. Positional accuracy during the sequence of static computer-assisted implant surgery in three alveolar ridge morphologies: an in vitro study. J Prosthodont. 2023;34:78. 37955870 10.1111/jopr.13798

[96] Jorba-García A, González-Barnadas A, Camps-Font O, Figueiredo R, Valmaseda-Castellón E. Accuracy assessment of dynamic computer-aided implant placement: a systematic review and meta-analysis. Clin Oral Investig. 2021;25(5):2479–94. 33635397 10.1007/s00784-021-03833-8

[97] Ozan O, Turkyilmaz I, Ersoy AE, McGlumphy EA, Rosenstiel SF. Clinical accuracy of 3 different types of computed tomography-derived stereolithographic surgical guides in implant placement. J Oral Maxillofac Surg. 2009;67(2):394–401. 19138616 10.1016/j.joms.2008.09.033

[98] Tahmaseb A, Wu V, Wismeijer D, Coucke W, Evans C. The accuracy of static computer-aided implant surgery: a systematic review and meta-analysis. Clin Oral Implants Res. 2018;29(Suppl 16):416–35. 30328191 10.1111/clr.13346

[99] Wu Y, Wang F, Fan S, Chow JK. Robotics in dental implantology. Oral Maxillofac Surg Clin North Am. 2019;31(3):513–8. 31103316 10.1016/j.coms.2019.03.013

[100] Wu XY, Shi JY, Qiao SC, Tonetti MS, Lai HC. Accuracy of robotic surgery for dental implant placement: a systematic review and meta-analysis. Clin Oral Implants Res. 2024;35(6):598–608. 38517053 10.1111/clr.14255

